# Post universal health coverage trend and geographical inequalities of mortality in Thailand

**DOI:** 10.1186/s12939-016-0479-5

**Published:** 2016-11-22

**Authors:** Suchunya Aungkulanon, Viroj Tangcharoensathien, Kenji Shibuya, Kanitta Bundhamcharoen, Virasakdi Chongsuvivatwong

**Affiliations:** 1 Epidemiology Unit, Faculty of Medicine, Prince of Songkla University, Songkhla, Thailand; 2 International Health Policy Program, Ministry of Public Health, Nonthaburi, Thailand; 3 Department of Global Health Policy, Graduate School of Medicine, University of Tokyo, Tokyo, Japan

**Keywords:** Geographical inequalities, Mortality, Thailand

## Abstract

**Background:**

Thailand has achieved remarkable improvement in health status since the achievement of universal health coverage in 2002. Health equity has improved significantly. However, challenges on health inequity still remain.This study aimed to determine the trends of geographical inequalities in disease specific mortality in Thailand after the country achieved universal health coverage.

**Methods:**

National vital registration data from 2001 to 2014 were used to calculate age-adjusted mortality rate and standardized mortality ratio (SMR). To minimize large variations in mortality across administrative districts, the adjacent districts were systematically grouped into “super-districts” by taking into account the population size and proximity. Geographical mortality inequality among super-districts was measured by the coefficient of variation. Mixed effects modeling was used to test the difference in trends between super-districts.

**Results:**

The overall SMR steadily declined from 1.2 in 2001 to 0.9 in 2014. The upper north and upper northeast regions had higher SMR whereas Greater Bangkok achieved the lowest SMR. Decreases in SMR were mostly seen in Greater Bangkok and the upper northern region. Coefficient of variation of SMR rapidly decreased from 20.0 in 2001 to 12.5 in 2007 and remained close to this value until 2014. The mixed effects modelling revealed significant differences in trends of SMR across super-districts. Inequality in mortality declined among adults (≥15 years old) but increased in children (0–14 years old). A declining trend in inequality of mortality was seen in almost all regions except Greater Bangkok where the inequality in SMR remained high throughout the study period.

**Conclusions:**

A decline in the adult mortality inequality across almost all regions of Thailand followed universal health coverage. Inequalities in child mortality rates and among residents of Greater Bangkok need further exploration.

## Background

Tackling health inequity is high on the current global and national agenda [1]. Equity is the cornerstone of the Sustainable development goals (SDGs,) entailing “leaving no one behind”. The overall SDGs call for healthy life for all ages, has positioning equity as a core, cross-cutting theme [2]. SDG 10 calls for actions to reduce inequality within and among countries, and Target 3.8 calls for the establishment of the universal health coverage (UHC) as a key instrument to achieve health goal with an equitable outcome. Thus, monitoring changes in disparities of health status, use of health services, and healthcare expenditure is essential for identifying gaps, and understanding which groups are being left behind, in order to implement effective interventions [3].

Since the achievement of UHC in Thailand in 2002, several evaluations have reported substantial improvement in the health status [4, 5], overall use of health services [6, 7], preventive activities [8], and reduction in catastrophic health expenditure and medical impoverishment [9, 10]. To our knowledge, there is only one study from Japan reporting the long-term impact of UHC on mortality; however, without assessment across geographical areas [11].

A recent study noted a continued decline in mortality rates in Thailand [12]. However, national average obscures intra-national differences and inequalities. Previous studies have noted significant regional variations in health outcome such as prevalence of chronic diseases [13–15], and their risk factors such as obesity [16] and hypertension [17]. Two pre-UHC studies reported sharp variations in all-cause and cause-specific mortality rates across geography but both studies were assessed before achievement of UHC [18, 19]. Whether the level of these health inequalities has been reduced following UHC has not been well documented. To fill the knowledge gap, we assessed the 14-year trend of geographical inequalities in mortality within different areas using vital registration data and tested the null hypothesis of different trends across these areas.

## Methods

### Data sources

Individual mortality data from all causes for years 2001 through 2014 were obtained from the national vital registration database, Ministry of Interior where cause of death was coded using the International Classification of Diseases, Tenth Revision (ICD-10) by the Bureau of Policy and Strategy, Ministry of Public Health. The 12 common deaths based on category of World Health Organization’s Mortality Tabulation list including tuberculosis (ICD10 codes A15-A19, B90), HIV/AIDS (B20-B24), liver cancer (C22), lung cancer (C33-C34), diabetes (E10-E14), ischemic heart disease (IHD) (I20-I25), stroke (I60-I69), pneumonia (J12-J18), chronic obstructive pulmonary disease (COPD) (J40-44), asthma (J45), cirrhosis (K74), and traffic injuries (V01-V04, V06, V09-V80, V87, V89, V99) were chosen. Mortality was credited to each district according to the individual’s registered place of residence. Mid-year population data of each districts for the period 2001-2014 was obtained from the Bureau of Registration Administration, Ministry of Interior.

Age- and sex-adjusted mortality rate and standardized mortality ratio (SMR) were calculated to measure mortality across time and population. The average Thai population age and sex structure during 2001-2014 was used as the standard population and further used as a weighting factor to calculate age- and sex-adjusted mortality rate. Geographic inequality in SMR across different areas and age groups was measured by the coefficient of variation (CV) at each year. Piecewise regression analysis was used to identify the time point of changing in trend.

Thailand was a lower middle-income country with a population of around 66 million during the study period. The country is divided into 77 provinces and 928 administrative districts. The population of these districts varies substantially, ranging from a minimum of 2411 to a maximum 521,651. To reduce the problem of difference in denominator, we regrouped the districts to show stable rates in areas with sparse population. Based on 2014 population, small districts within each province was merged with larger and adjacent districts (or groups of already merged districts) and the resulting areas were called “super-district”. The definition of a super-district is a district or group of districts that contains a minimum population of 100,000 persons. We applied the method outlined in Babcock’s study to group districts into super-districts [20]. As the population in the districts and number of administrative districts also changed over the 14-year study period. The district population sizes in 2014 were used to construct the super-districts. Before merging, the mean (standard deviation) population of all districts was of 68,644 (55,233). After merging, a total of 331 super-districts out of the total 928 administrative districts were formed with population ranging from 100,849 to 521,651, and a mean (standard deviation) of 195,524 (73,805). Our preliminary analysis revealed that the distribution of all-cause SMRs at the super-district level was normal. Parametric statistics were therefore used throughout the analysis. Mixed effects modeling was used to test whether trends (slope) of SMR across super-districts were equal. All data analysis was undertaken and graphical displays created using SAS software version 9.4.

## Results

A total of 5,539,154 deaths were reported in the death registration database during the 14-year study period. The all cause age-adjusted mortality rate decreased from 672.12 per 100,000 population in 2001 to 574.94 in 2014, with an average annual decline of 6.07 per 100,000 (95% CI: 3.15–8.99).

**Fig. 1:**
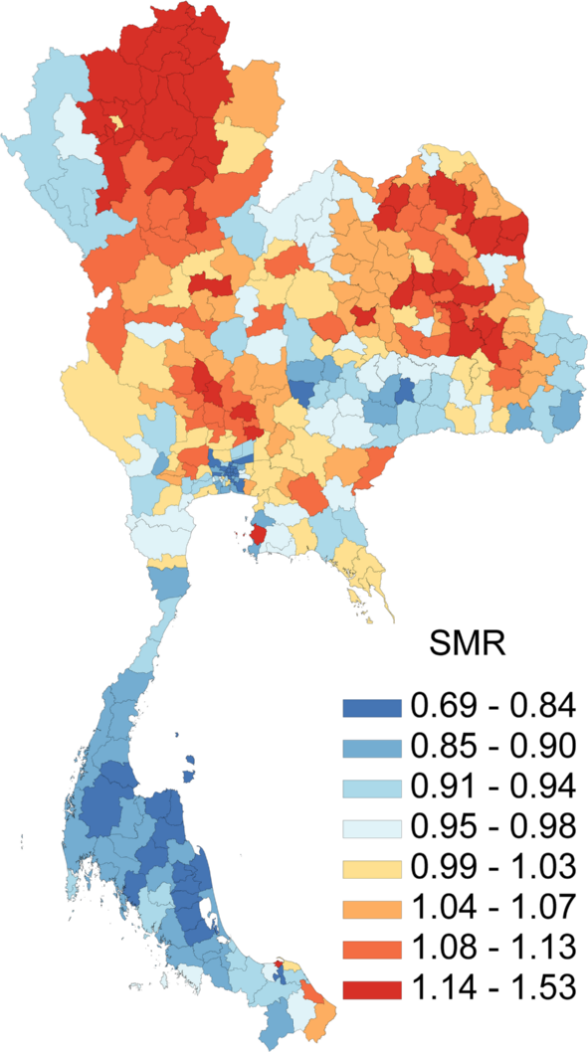
Geographical distributions of average annual standardized mortality ratio by super-district, 2001-2014

**Fig. 2:**
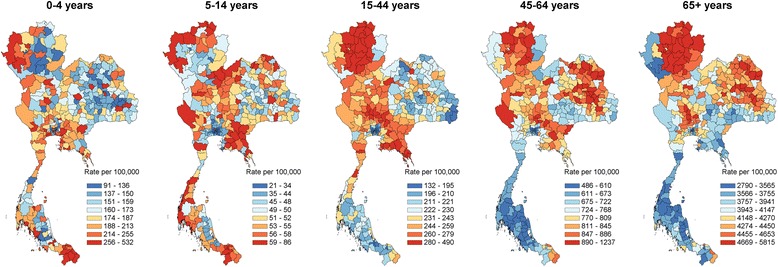
Geographical distributions of average annual age-specific mortality rate, 2001-2014

Figure 1 presents the geographical distribution of average annual SMR during 2001–2014 across super-districts of Thailand. Clustering of high SMR was largely confined to certain areas of the northern and upper northeast regions whereas in Greater Bangkok (Bangkok, Prathumtani, Nonthaburi, Samutprakan, Samutsakhon, Nakornprathom provinces), and the southern region was relatively low. Figure 2 shows the geographical distribution of age specific mortality rates for various age groups. Child mortality was obviously high along Myanmar and Malaysia borders.

**Fig. 3:**
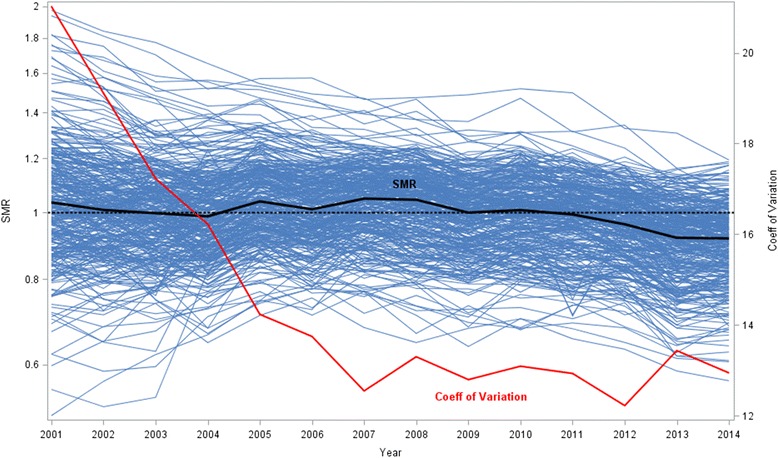
Trend of standardized mortality ratio over time, 2001-2014

Figure 3 displays trend of SMR for each super-district demonstrated by spaghetti plot; the median SMR is plotted as a black line and CV shown in red. The CV decreased rapidly during the first half of the study period (from 20.0 in 2001 to 12.5 in2007) but remained relatively constant in recent years (2008–2014). Piecewise linear regression confirmed this visual impression with a slope of -1.31 unit per year between 2001 and 2007 and increased by only 0.09 per year between 2008 and 2014. Furthermore, mixed effects modeling with year as fixed effects revealed that slops of the super-districts were not equal but should be treated as significant random variables (*p* < .0001). This indicated different trends of mortality rates.

**Fig. 4:**
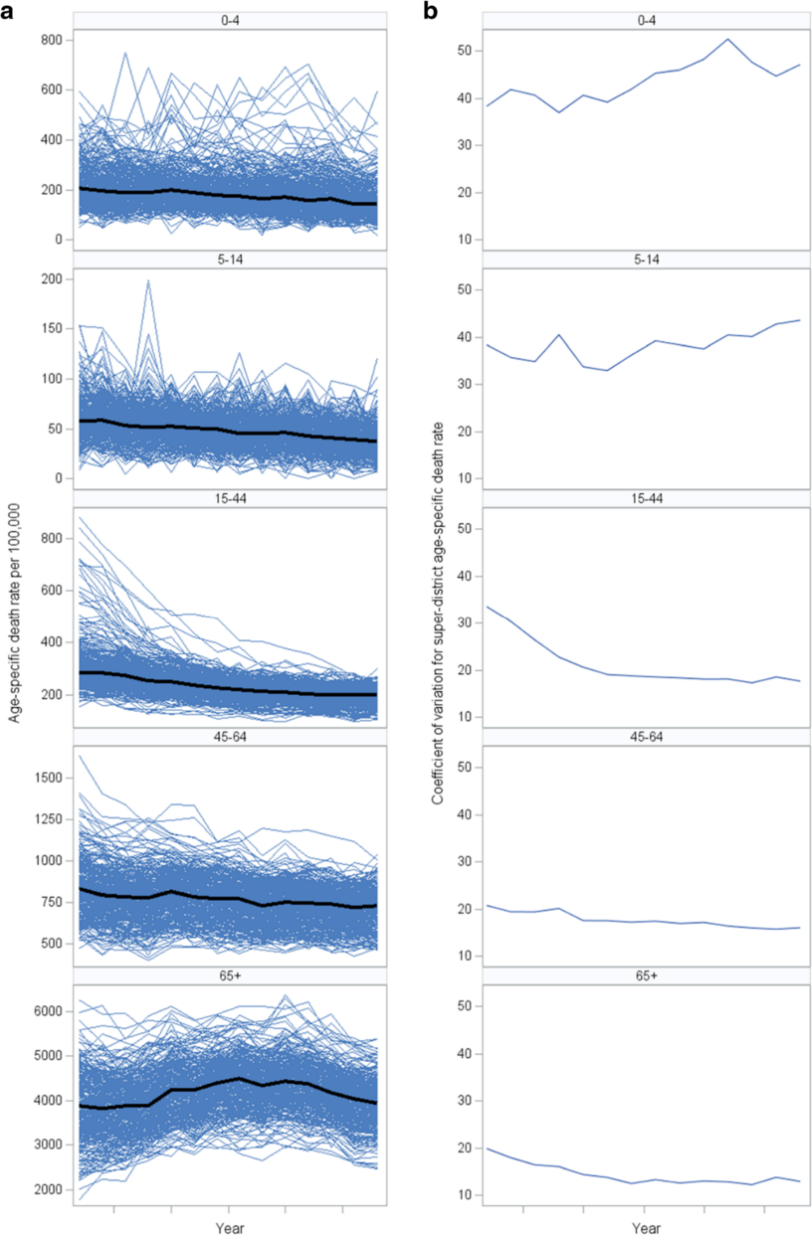
Trend in super-district death rate and CV by age group, 2001-2014. (**a**) Age specific death rate per 100,000 (**b**) Coefficient of variation for super-district age specific death rate

**Fig. 5:**
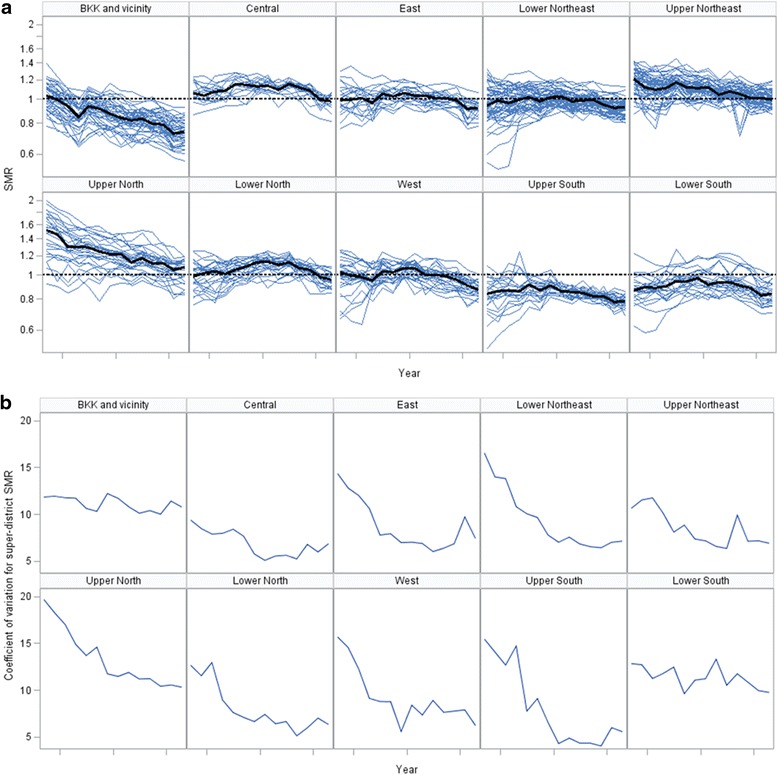
Trend in super-district level standardized mortality ratio and coefficient of variation by region, 2001-2014. (**a**) Age specific death rate per 100,000 (**b**) Coefficient of variation for super-district age specific death rate

Figure 4, Panel A shows that the mortality rates decreased in all age groups except 65+, where it went up and then down in a curvy way. In Panel B, the CV declined in age groups 15–44 and 65+ years while increased in age group 0–4 years. Figure 5 shows that mortality rates declined steadily and most sharply in Greater Bangkok and the upper northern region. The initial high SMR in 2001 in the upper northern region had steadily declined to the average national level among other regions, where their SMRs were relatively stable since the beginning of the study period. In Fig. 5b, there was a declining trend of mortality inequality in all regions except Greater Bangkok. The upper southern part had the highest reduction of mortality variation (65.93% reduction in CV for SMR), followed by west (60.54%), lower northeast (57.11%), lower north (50.20%), and upper north (47.71%).

**Fig. 6:**
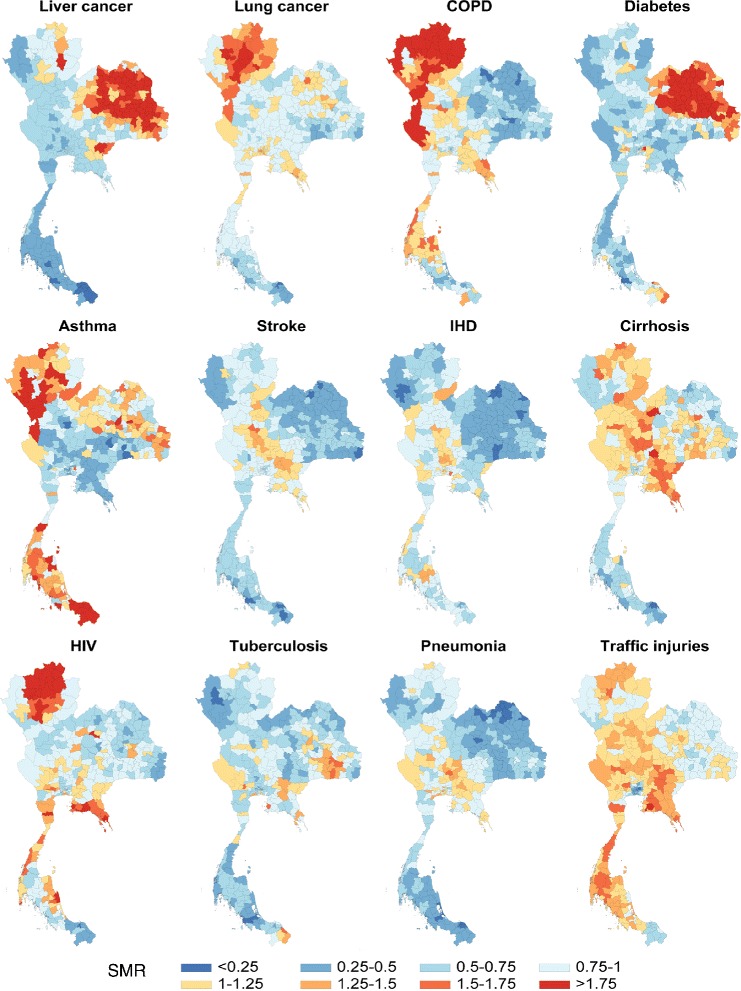
Geographical distributions of super-district average annual cause-specific standardized mortality ratio, 2001-2014

The geographical distributions of cause-specific mortality for the 12 selected causes of death are shown in Fig. 6. The northern region had higher rate of death from HIV/AIDS, COPD, lung cancer, and asthma compared to the other regions. The upper northeast region had relatively high rates of death from liver cancer and diabetes. Compared with the upper northeast, the lower northeast had lower mortalities from liver cancer, diabetes, and others except tuberculosis. The southern part had relatively low mortality from most diseases except asthma and traffic injuries. Deaths from stroke, IHD, tuberculosis and pneumonia were geographically homogeneous though the rates were relatively higher in the central region. For traffic injuries, super-districts having high SMR seem to be spread throughout most region, except the northeast. Similar to all-cause mortality, the distribution patterns for each of 12 selected conditions were clustered.

**Fig. 7:**
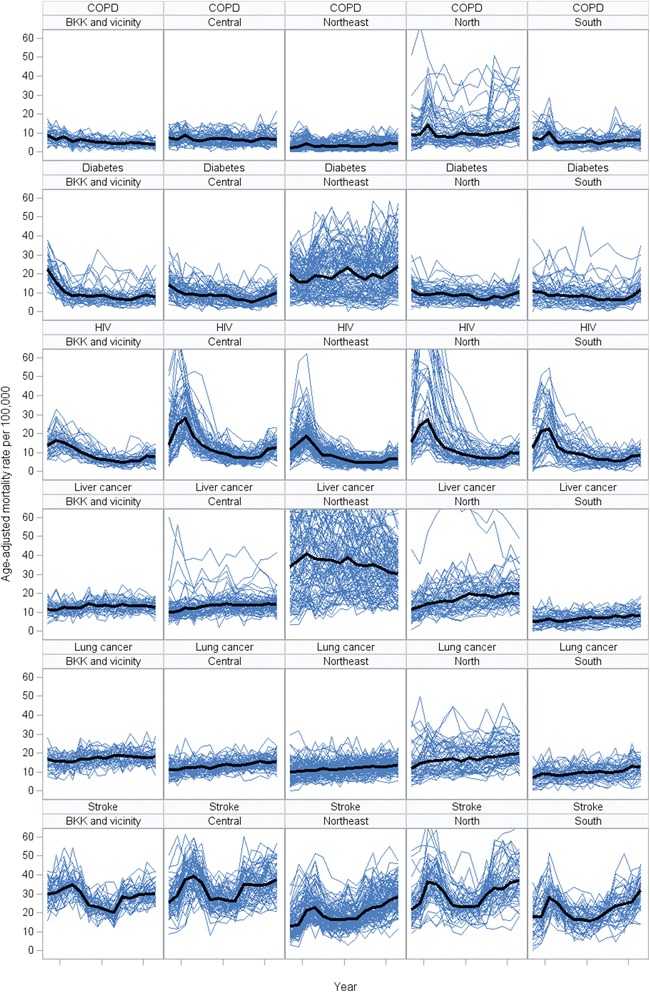
Trend in super-district cause-specific mortality rates, six selected conditions, by region, 2001-2014

**Fig. 8:**
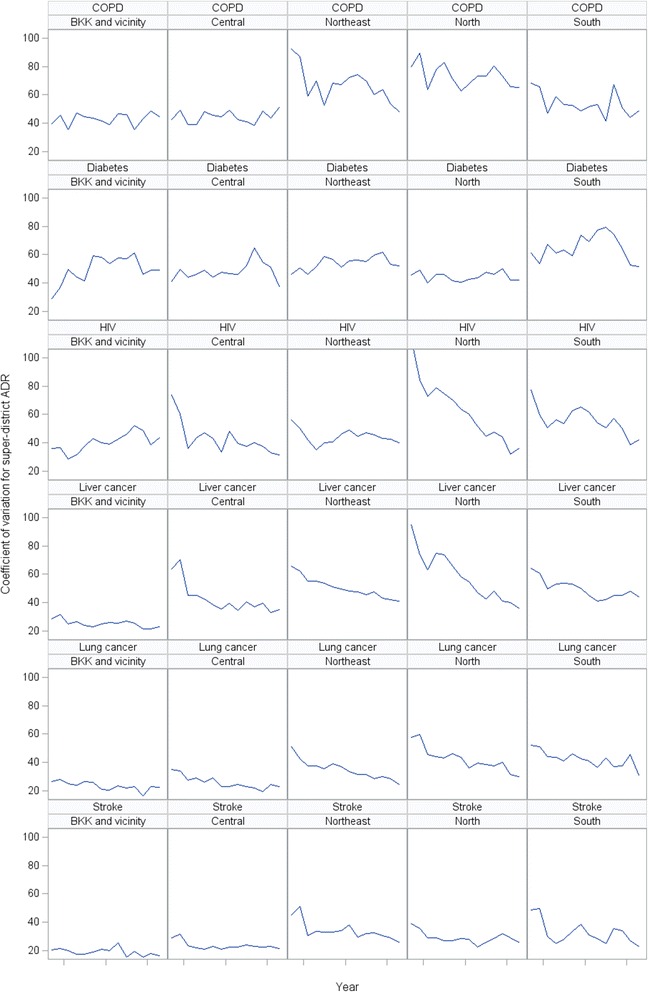
Trend in super-district coefficient of variation of cause-specific mortality rates, six selected conditions, by region, 2001-2014

Figure 7 show trends of mortality rates of six (out of 12) selected disease groups. Both lung and liver cancer mortalities showed an increasing trend in all regions. COPD and diabetic mortality rates remain relatively stable in most regions whereas stroke showed a long term upward mortality trend. Trends of HIV mortality declined, particularly in the upper north region. Figure 8 displays trends in inequality of cause-specific mortality by region. The inequalities of most diseases mortalities have either declining or stable trends in all regions except diabetes and HIV/AID mortalities in Greater Bangkok had increasing inequality trends.

## Discussion

The overall mortality in Thailand have steadily declined since universal health coverage was achieved in 2002. Geographical inequalities in mortality rapidly decreased in the first half of the study period and has since become stable. A decline in the inequality gaps was seen in adults but gaps remained high among children. The reduction in mortality was mostly seen in Greater Bangkok, a low mortality region, and the upper north, a region with high mortality. Most regions had a declining trend in mortality inequality, while in Greater Bangkok, though the rate was low, the inequality remained high. Variation in mortality rates with regional and sub-regional clustering was observed for several causes of death. For example, a high mortality rate of liver cancer and diabetes was seen in the upper northeast. COPD and lung cancer were high in the upper northern region. Inequalities for most chronic diseases have declined or stabilized in all regions except for diabetes and HIV/AID which both had increasing trends in Greater Bangkok.

A steady decline in mortality rates have been documented worldwide [12, 21–24]. A combination of many factors including economic growth, poverty reduction, and a variety of contributing factors such as literacy rates, particular among females, and education level have had a positive influence on these health achievements in the past few decades. Better nutrition and sanitation are also major contributing factors. Our study showed that disparity in mortality reduced rapidly between 2001 and 2007 and stabilized in subsequent years. This declining inequality trend of overall mortality was mostly influenced by the reduction inequality of HIV mortality in person aged 15–44 years in the north region of Thailand. Deaths from HIV was the number one ranked cause of death among the adults [25] and was concentrated more in the northern region where is known to be the epicenter of the HIV epidemic in Thailand [26, 27]. Reduction in HIV mortality in this working age group followed the successful HIV prevention program (in particular vertical prevention from mother to child) and high coverage of universal access to antiretroviral treatment (ART) for HIV may explain all these trends [28, 29] The UHC extended the insured population from about twenty-five million (40% of the population) in 2001 to above fifty-nine million (95.5%) in 2004 [30]. The reduction in inequality documented here was partly due to the improvement in the benefits package and full population coverage under the universal healthcare policy.

The recent stagnant reduction in health inequality highlights the needs for greater efforts on further reduction. Inequality in child mortality has been high and sustained throughout the study period. High mortality of children aged under-five years in areas close to Myanmar (where there are a large number of ethnic minority groups, stateless people and hard to reach mountainous areas) and Malaysia (in particular among the Muslim communities) highlights a real challenge to improve access to health services among children in these sub-regions. Although universal access to maternal and child health services is well founded in Thailand [31], our results indicate the need to emphasize better care in the marginalized communities [32, 33]. The overall mortality rate has been falling in most regions. The largest reductions have been observed in Greater Bangkok and the north. The huge decline in Greater Bangkok could be due to rapid improvements in the standard of living due to economic prosperity. The case of sharp reduction in the north has already been described above. However, the consistently high level of inequality, and low mortality in Greater Bangkok suggests a need for further investigation.

Our findings of geographical clustering of all-cause mortality and cause-specific mortality are consistent with those of previously published papers that examined geographical variation of mortality in Thailand using national vital registration data for the 2000 period [18, 19]. High overall mortality was observed in the northeastern and northern regions. This is probably due to higher poverty levels in these two regions [34]. Health workforce density was found to be poorer than other affluent areas such as Bangkok Metropolis [35–37].

Clustering of high mortality from liver cancer in the upper northeast can be explained mainly by the extremely high prevalence of chronic liver fluke infestation, the major risk factor of cholangiocarcinoma [38]. The local community habit of eating uncooked cyprinoid fish infested with *Opisthorchis viverrini* results in high prevalence of cholangiocarcinoma in northeastern Thailand [39]. Clustering of high lung cancer, COPD, and asthma mortality in the north may reflect ambient air pollution. However, slightly high mortality from asthma in the southern region needs further investigations. Although the southern region has the highest smoking rate in the country [40], smoking alone cannot fully explain the clustering of respiratory deaths. Variation in deaths for stroke and IHD by region were also evident, with the greatest rates found in the lower northern, central, and eastern regions which are the industrial zones. Areas with low cardiovascular mortality tended to be in the northeast region. The high mortality of diabetes mellitus is another major public health problem in the northeastern region. Many studies found that the prevalence of diabetes was higher in urban than in rural areas [41, 42]. The higher rates of mortality for diabetes in the northeast, where the majority of the population reside in rural areas highlight the need for subnational assessment for associated risk factor, and quality of health care.

Districts with sparse populations were combined into super-districts to prevent the creation of unstable mortality rates. However, super-district is an areal unit, delineated by larger administrative district boundaries rather than natural features or cohesive groups and as such is subject to the Modifiable Areal Unit Problem (MAUP) [43].

Despite the fact that up to 98% of total deaths are certified, the quality of mortality statistics was considered poor because a large proportion of registered death are classified as being due to ill-defined conditions [44, 45]. One reason for the poor quality of recorded causes of death was that about 60% of the deaths occurred outside hospitals and thus were recorded and coded by nonmedical personnel. While this issue may have a potential influence on cause-specific mortality rates, it may have little influence on the overall trends.

Although it is clear that Thailand’s UHC was followed by a sharp reduction in mortality inequality, this cross-sectional study cannot elucidate causality. There are several possible determinants of mortality inequality including demographic, socio-economic, geographic, and health system variables that were not included in this analysis, thus the ability to explain this inequality is limited.

## Conclusions

In conclusion, during the 14 years of UHC scheme in Thailand, a steady decline of mortality was observed with varying degrees of reduction in inequality across age groups, diseases and geographical areas. Persistent disparities in mortality outcomes in children and in the capital city are still a concern. More effort is needed to further reduce these inequalities. Findings from regular monitoring of mortality inequality can contribute to specific policy interventions.

## Abbreviations

COPD: Chronic obstructive pulmonary disease
CV: Coefficient of variance
ICD-10: International classification of diseases tenth revision
IHD: Ischemic heart disease
SDG: Sustainable development goals
SMR: Standardized mortality ratio
UHC: Universal health coverage
